# Suppression of kidney fibrosis by cGMP-dependent protein kinase I

**DOI:** 10.1186/1471-2210-11-S1-P62

**Published:** 2011-08-01

**Authors:** Elisabeth Schinner, Armin Kurtz, Franz Hofmann, Jens Schlossmann

**Affiliations:** 1 Pharmakologie und Toxikologie, Universität Regensburg, Germany; 2 Physiologie, Universität Regensburg, Germany; 3 Carvas-Zentrum, TU München, Germany

## Background

cGMP is synthesized via nitric oxide- or natriuretic peptide-stimulated guanylyl cyclases and exhibits pleiotropic regulatory functions also in the kidney. Hence, the integration of cGMP signaling via cGMP-dependent protein kinases (cGK) might play a critical role for renal physiology. Both isozymes were detected in arterioles, mesangium and within the cortical interstitium. In contrast to cGKIα, the β isoform was not detected in the juxtaglomerular apparatus and medullary fibroblasts.

## Results

Here, we examined the function of cGKI in the renal interstitium emphasizing a functional differentiation of both isoforms. The interstitium exists mainly of fibroblasts playing a prominent role in the interstitial fibrosis. Accordingly, cGKI could also be involved in this pathophysiological process. Therefore, we studied whether cGKI influences renal fibrosis by application of cGMP increasing YC-1 or ISDN and by using mutant mice. The kidney-fibrosis was induced by unilateral ureter obstruction (UUO).

## Conclusion

Administration of ISDN showed significantly antifibrotic effects in wt- but not in αSM-rescue mice. Also tg-tg mice which express more cGKIα developed significantly less fibrosis than wt mice. Moreover, mRNA- and protein expression of cGKIβ was fewer influenced by fibrosis than cGKIα. Accordingly, our results indicate that cGMP acts primarily via cGKIα as an important suppressor of kidney fibrosis.

